# Normative Database and Determinants of Retinal and Choroidal Vessel Density in Tibetan Children

**DOI:** 10.3390/children13020284

**Published:** 2026-02-19

**Authors:** Zhaojun Meng, Yao Yao, Lei Li, Weiwei Chen, Jing Fu

**Affiliations:** Beijing Tongren Eye Center, Beijing Tongren Hospital, Capital Medical University, Beijing Key Laboratory of Ophthalmology & Visual Sciences, Beijing 100730, China; mengzhaojun1985@126.com (Z.M.); stellayao2025@163.com (Y.Y.); yk9157@126.com (L.L.); wuren508@163.com (W.C.)

**Keywords:** retinal vessel density, children, Tibetan, choroidal thickness, choriocapillaris

## Abstract

**Highlights:**

**What are the main findings?**

- Foveal retinal vessel density in Tibetan children is positively correlated with systemic oxygen saturation.
- The retinal microcirculation reflects adaptation to chronic hypoxia.

**What are the implications of the main findings?**

- This study provides normative OCTA data for this unique pediatric population and identifies systemic oxygen saturation as a novel determinant of foveal vascular density.
- This study positions retinal vascular metrics as a potential non-invasive biomarker for systemic oxygen homeostasis and high-altitude adaptation.

**Abstract:**

Background/Objectives: This study investigates the normative data and determinants of retinal and choroidal vessel density (VD) in Tibetan children using optical coherence tomography angiography (OCTA). Methods: This study recruited students from primary schools in Lhasa who underwent OCTA encompassing VD in the superficial capillary plexus (SCP) and the deep capillary plexus (DCP) and choriocapillaris (CC) in the macular region, as well as refractive status, axial length, and systemic examinations. Results: This study included a total of 645 children who met the criteria. The results showed that VD in the fovea was significantly higher in the SCP than in the DCP, while CC had the highest VD in the fovea. Correlation analysis revealed strong correlations in VD among all quadrants of the SCP, DCP, and CC, as well as significant correlations between corresponding regions of the SCP and DCP. VD showed no significant association with age, sex, axial length, or spherical equivalent. Foveal VD in both the SCP and DCP was positively correlated with oxygen saturation. No consistent correlation was found between choroidal or retinal thickness and VD in any layer. Conclusions: The identified link between systemic oxygen saturation and foveal vascular density offers a novel perspective on human adaptation to chronic hypoxia, positioning the retinal microcirculation as a sensitive indicator of systemic oxygen homeostasis.

## 1. Introduction

Lhasa, located in the middle of the Qinghai–Tibet Plateau at an altitude of 3650 m, is one of the highest cities in the world. The high-altitude environment, particularly its defining feature of chronic hypobaric hypoxia, poses unique challenges to human physiological systems and induces a series of complex adaptive changes [1]. The eye, as a highly metabolically active and densely vascularized organ, contains a retina that is particularly sensitive to alterations in blood oxygen supply. Consequently, the retinal vascular system is regarded as a crucial window into systemic microcirculatory status [2,3]. In the fields of high-altitude medicine and ophthalmic research, investigating the retinal vascular characteristics of long-term high-altitude residents is of significant value for understanding the mechanisms of hypoxia adaptation and their potential pathophysiological implications.

Childhood represents a critical period for the development of the visual system and other organs. During this stage, adequate oxygen supply is essential for the normal development and functional maturation of the retina, particularly the macular region [4,5]. As the area responsible for the highest visual acuity, the macula—with its dense array of photoreceptors and complex neuronal networks—relies on a sophisticated three-dimensional vascular network formed by the underlying choriocapillaris and the superficial and deep retinal capillary plexuses [6]. Current quantitative studies of the retinal vasculature have predominantly focused on adult populations from low-altitude regions, especially in the context of systemic vascular diseases such as hypertension and diabetes [7,8]. However, a substantial knowledge gap remains regarding the developmental patterns, vascular density distribution, and structural characteristics of the retinal vasculature in healthy children native to high-altitude regions under chronic hypoxia. This gap limits our ability to comprehensively assess the potential long-term effects of high-altitude environments on visual development and ocular health in children.

Previous studies of the high-altitude retina have largely relied on color fundus photography to analyze retinal arteriolar and venular diameters in two-dimensional images or have focused on clinical conditions such as high-altitude retinopathy associated with polycythemia [9,10]. These approaches do not allow precise, layer-specific quantification of the three-dimensional density of the macular microvasculature. The advent of optical coherence tomography angiography (OCTA) has revolutionized retinal microvascular research. OCTA enables noninvasive, rapid, high-resolution, layer-specific imaging and quantitative analysis of the superficial capillary plexus, deep capillary plexus, and choriocapillaris, providing detailed morphological parameters such as vessel density, perfusion area, and foveal avascular zone metrics [11,12].

Lhasa, situated on the Tibetan Plateau at approximately 3650 m above sea level, where the atmospheric partial pressure of oxygen is about 60% of that at sea level, provides an ideal natural laboratory for studying the effects of chronic hypoxia in humans. This study aims to use OCT and OCTA imaging to systematically characterize the distribution patterns and normative ranges of macular retinal vessel density in the superficial and deep capillary plexuses in healthy children native to Lhasa. Furthermore, we will examine the associations between retinal microvascular parameters and key ocular biometric parameters (such as axial length, corneal curvature, and refractive status), as well as important systemic factors (including age, sex, height, weight, body mass index, blood oxygen saturation, and hemoglobin levels).

We anticipate that this study will provide the first comprehensive map of macular retinal vessel density in healthy children residing at high altitude. The findings will not only fill a critical data gap in this field and enhance our understanding of retinal microcirculatory development in high-altitude children but may also offer novel microcirculatory indicators for evaluating physiological adaptation to hypoxia. Moreover, the baseline data established in this study will provide a solid theoretical foundation for future longitudinal research and the early identification of potential ocular health risks associated with high-altitude environments in children.

## 2. Materials and Methods

### 2.1. Study Protocol

The Lhasa Childhood Eye Study is a school-based epidemiological cohort designed to investigate ocular development and the incidence of eye diseases among primary school children in Lhasa, China. At baseline in 2019, a total of 1856 students from seven primary schools were enrolled through stratified random cluster sampling and were subsequently examined annually. Each participant underwent a comprehensive ocular and basic systemic evaluation in accordance with the study protocol. In October 2021, an additional 663 healthy children were randomly recruited for OCTA imaging. This study adhered to the Declaration of Helsinki and was approved by the Ethics Committee of Beijing Tongren Hospital, Capital Medical University (No. TRECKY2019-058). Written informed consent was obtained from each participant and/or their legal guardian.

### 2.2. Ocular Examinations and Definitions

All participants underwent a comprehensive bilateral ophthalmic evaluation, including measurements of uncorrected and best-corrected visual acuity (BCVA) (250300; Good-Lite, Elgin, IL, USA), stereoacuity (S0001, STEREO, Los Angeles, CA, USA), ocular dominance, slit-lamp biomicroscopy (SL-3G, Topcon, Tokyo, Japan), tonometry (CT-800, Topcon, Tokyo, Japan), ocular motility assessment, autorefraction before and after cycloplegia, axial length measurement (IOLMaster, ZEISS, Oberkochen, Germany in 2021; Lenstar, Haag-Streit, Bern, Switzerland in 2019), OCT, and retinal photography. Height, weight, blood pressure, heart rate, and blood oxygen saturation (SpO_2_) were also recorded.

Axial length (AL), intraocular pressure, and refractive measurements were averaged from three consecutive readings. The spherical equivalent (SE) was calculated as the spherical power plus half of the cylindrical power, based on cycloplegic autorefraction results. A trained technician used swept-source OCT and OCTA (DRI OCT Triton-1, Topcon, Tokyo, Japan) to measure retinal and choroidal thickness. Because choroidal thickness varies with circadian rhythm and cycloplegia, all examinations were conducted prior to cycloplegia, between 1:00 p.m. and 4:00 p.m. [13].

### 2.3. OCTA Measurements

For OCTA vessel density (VD) assessment, we used the Triton-1 to perform imaging, which operates at a central wavelength of 1050 nm, with an axial resolution of 8 μm, a transverse resolution of 20 μm, and a scanning speed of 100,000 A-scans per second. All images were obtained by a well-trained examiner, with participants seated and fixating on a target after pharmacologic dilation. Only images of sufficient quality (signal strength index > 40) were included. A 6 × 6 mm “angio macula” scan was automatically centered on the fovea.

The OCTA ratio analysis algorithm, which preserves full-spectrum data and axial resolution, was applied. VD was defined as the proportion of perfused vessel area within the measured region and was quantified using adaptive threshold binarization to minimize uneven image brightness.

**Figure 1:**
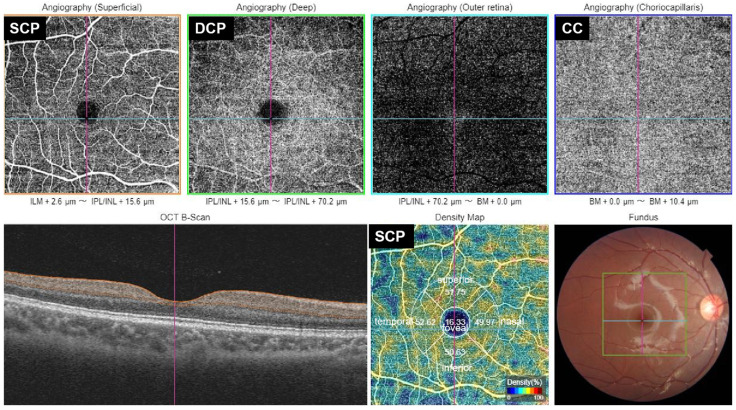
Representative 6 × 6 mm OCTA images obtained by Topcon DRI OCT Triton-1 device. Macula was regionally measured in the whole ETDRS grid area, which comprised 2 concentric rings: 1 mm fovea center, 1 to −3 mm parafoveal area. ILM, Inner Limiting Membrane, IPL, Inner Plexiform Layer, INL, Inner Nuclear Layer, BM, Bruch’s Membrane.

VD was quantified in five subfields—the fovea and four parafoveal quadrants (inferior, superior, nasal, and temporal). These were identical to the Early Treatment Diabetic Retinopathy Study (ETDRs) subregion. Vessel density was obtained after verification of segmentation accuracy and application of projection-removal algorithms for images. For macular VD (Figure 1), the software automatically segmented the superficial capillary plexus (SCP), deep capillary plexus (DCP), and choriocapillaris (CC) in each region (version 2014.2.0.65). By default, the SCP extends from 2.6 μm below the internal limiting membrane to 15.6 μm beneath the inner plexiform layer (IPL); the DCP from 15.6 to 70.2 μm below the IPL; and the CC from Bruch’s membrane to 10.4 μm beneath it.

All examinations were performed in both eyes. Given the high interocular agreement (r = 0.95), only data from the right eye of healthy participants were included in the analysis [14].

### 2.4. Statistical Analysis

Data are presented as the mean ± standard deviation for continuous variables and as the median (interquartile range) for non-normally distributed variables. Statistical analyses were conducted using IBM SPSS Statistics (version 27.0; IBM Corp., Chicago, IL, USA). Correlations between macular VD and systemic factors were assessed using binary correlation analysis. Differences in VD between the SCP and DCP were evaluated using paired *t*-tests. Multivariate linear regression analysis was performed to identify factors associated with higher or lower macular VD. Statistical significance was defined as *p* < 0.05.

## 3. Results

Due to poor cooperation and language communication difficulties in some children, only a subset of the cohort underwent and successfully completed the OCTA examination. After excluding images of poor quality or with significant abnormalities, 645 children met the inclusion criteria. The mean age of the participants was 8.57 ± 0.50 years; 47.8% were female, and 98.3% were Tibetan. The demographic and clinical characteristics of the study population are presented in Table 1. Significant correlations in vessel density (VD) were observed between the left and right eyes across all sectors of the superficial capillary plexus (SCP), deep capillary plexus (DCP), and choriocapillaris (CC). Moreover, no statistically significant interocular differences in VD were detected.

The mean foveal VDs were 17.7% ± 4.6% (range, 5.6–31.8) in the SCP and 13.0% ± 4.4% (range, 3.6–35.1) in the DCP, corresponding to the foveal region; VD was significantly higher in the SCP than in the DCP (*p* < 0.001). The mean parafoveal VDs in the SCP were 47.3% ± 2.3%, 46.3% ± 2.9%, 46.6% ± 2.3%, and 48.6% ± 2.2% in the superior, inferior, nasal, and temporal quadrants, respectively. In the DCP, the corresponding values were significantly higher at 49.9% ± 2.8%, 47.7% ± 3.4%, 47.9% ± 3.0%, and 47.8% ± 3.0% (*p* < 0.001 for all quadrants). The mean foveal VD in the CC was 55.1% ± 3.5% (range, 21.2–63.9), which was significantly higher than that in the SCP and DCP across all quadrants (*p* < 0.001).

Univariate correlation analysis demonstrated strong correlations in vascular density among the four parafoveal sectors within the SCP, DCP, and CC. Significant correlations were also observed between corresponding sectors (superior, inferior, nasal, temporal, and foveal) of the SCP and DCP. No significant associations with age or sex were identified in any sector of the SCP, DCP, or CC. Furthermore, no significant correlations were found between axial length (AL) or spherical equivalent (SE) and VD in any sector. However, in the foveal region of both the SCP and DCP, VD was positively correlated with blood oxygen saturation. No significant correlations were detected between VD in any layer and other systemic parameters, including blood pressure, heart rate, height, or weight (Table 2).

In the multivariate regression analysis, after adjusting for age, sex, axial length (AL), and spherical equivalent (SE), foveal VD in both the SCP and DCP remained significantly and positively associated with blood oxygen saturation. However, this association was not observed for CC VD (Table 3).

Regarding ocular parameters, choroidal and retinal thicknesses were not significantly correlated with VD in any sector of the CC, SCP, or DCP. Although isolated correlations between VD and thickness were identified in a few scattered regions, these findings were not consistent across corresponding sectors. No significant association between vascular density and thickness was observed, including in the foveal region (Table 2).

## 4. Discussion

This study provides a comprehensive quantitative analysis of the macular microvasculature in a large cohort of healthy children native to high altitude using OCTA. Our principal findings are threefold. First, we established normative vessel density (VD) data for the SCP, DCP, and CC in this unique pediatric population. Second, we identified a significant and independent positive association between blood oxygen saturation and foveal VD in both the SCP and DCP, a correlation not observed in the CC or in the parafoveal regions. Third, retinal and choroidal microvascular densities demonstrated remarkable independence from other demographic, ocular, and systemic parameters, including age, sex, axial length, refractive error, and blood pressure.

The normative VD values reported herein are essential for future comparative investigations. The symmetry between left and right eyes and the strong intersector correlations within each capillary layer confirm the reproducibility of OCTA measurements and suggest coordinated developmental regulation of the macular microvasculature. The significantly higher VD in the parafoveal DCP compared with the SCP is consistent with established anatomical knowledge of the denser capillary network in the deep plexus, which primarily supplies the inner nuclear layer [15]. Similarly, the high VD of the CC underscores its function as a high-flow, fenestrated vascular bed responsible for the metabolic support of the retinal pigment epithelium and photoreceptors. The sparse vasculature in the central fovea reflects the need to maintain optical clarity and reliance on the underlying choriocapillaris for metabolic support [15]. Collectively, these findings establish a normative framework that may serve as a reference for detecting early microvascular alterations indicative of pathological processes.

The robust correlations among parafoveal sectors within and across layers highlight the interdependence of macular microvascular networks. Such coordinated variation may represent early manifestations of layered autoregulatory mechanisms that maintain retinal homeostasis [16]. Understanding these relationships is critical, as subtle dysregulation in childhood may predispose individuals to later susceptibility to retinal diseases, including myopia, diabetic retinopathy, and ischemic conditions.

The most salient finding of this study is the specific positive correlation between blood oxygen saturation and foveal VD in the SCP and DCP. In the context of chronic hypobaric hypoxia, this relationship may reflect an adaptive microvascular response. The fovea, devoid of intrinsic retinal vessels and reliant on the underlying CC and surrounding parafoveal capillaries of the SCP and DCP, represents a metabolic “hotspot” with high oxygen demand. In a reduced-oxygen environment, children with relatively higher blood oxygen saturation may exhibit more efficient oxygen uptake, transport, or utilization. This systemic advantage could translate locally into reduced compensatory vasodilation or neovascular drive, potentially manifesting as a less dense—but more efficiently perfused—capillary network in this critical region for high-acuity vision [17]. Conversely, children with lower oxygen saturation may experience a stronger hypoxic stimulus, leading to increased vascular endothelial growth factor (VEGF) expression and subsequent capillary remodeling or proliferation, thereby increasing VD as a compensatory response [18]. The confinement of this association to the foveal region further underscores its unique metabolic vulnerability and regulatory precision.

The absence of an association between CC VD and blood oxygen saturation is equally informative. It suggests that regulatory mechanisms governing retinal (SCP and DCP) and choroidal circulations are distinct. The choroidal circulation is highly autonomous, predominantly regulated by the autonomic nervous system and less responsive to local metabolic stimuli than the autoregulated retinal circulation. Our findings imply that chronic hypoxia at high altitude does not substantially alter the morphometric density of the choriocapillaris as measured by OCTA, or that compensatory responses occur through mechanisms such as changes in blood flow velocity or volume, which are not captured by static VD metrics [19].

The lack of association between VD and factors such as age, axial length, and spherical equivalent within this narrow age cohort is noteworthy. Some low-altitude pediatric studies have reported reductions in peripapillary VD with increasing age and axial length [20,21]. Our findings suggest that within this limited age range—during which structural and refractive development remains ongoing—macular microvascular perfusion is relatively stable. This stability indicates that the foundational architecture of the retinal microvasculature may be largely established by early childhood. Similarly, the absence of correlations with axial length or refractive status argues against a direct influence of early globe expansion on macular perfusion. These results challenge assumptions regarding the interplay between ocular biomechanics and retinal blood supply and suggest that microvascular vulnerability in myopia may emerge later, as axial elongation progresses [22]. The predominant influence of the high-altitude environment may overshadow subtler variations associated with normal ocular growth in this population, reinforcing the notion that normative data from low-altitude populations cannot be directly extrapolated to high-altitude natives [23].

Equally important is the absence of consistent associations between retinal or choroidal thickness and VD. Although both tissues undergo continuous developmental refinement during childhood, our findings indicate that structural growth and microvascular density may evolve along largely independent trajectories. This apparent dissociation challenges the traditional assumption that increased tissue thickness corresponds to greater vascular density [24]. Instead, structural and perfusion parameters may provide complementary but distinct insights into ocular development. Longitudinal studies may clarify whether subtle mismatches between thickness and perfusion precede pathological alterations.

Collectively, these findings enhance our understanding of macular vascular maturation in children residing at high altitude, a population exposed to chronic hypoxia. The strong association between systemic oxygen saturation and foveal perfusion may reflect adaptive physiological mechanisms in response to the hypobaric environment of the Tibetan Plateau. These adaptations have broader implications for interpreting pediatric OCTA metrics across different altitudes and populations.

Several limitations should be acknowledged. First, the cross-sectional design precludes causal inferences regarding the relationship between blood oxygen saturation and VD. Longitudinal studies are required to characterize the evolution of these microvascular parameters throughout childhood and adolescence. Second, although OCTA provides high-resolution structural imaging, it does not directly measure blood flow dynamics. Complementary functional assessments, such as retinal oximetry or laser Doppler flowmetry, would offer a more comprehensive evaluation of oxygen delivery and consumption. Third, despite adjustment for major confounders, unmeasured genetic or environmental factors specific to the Tibetan population may influence both oxygen saturation and retinal vascular development.

In conclusion, this study delineates the distinctive characteristics of the macular microvasculature in children native to high altitude. The identified association between systemic oxygen saturation and foveal VD offers novel insight into human adaptation to chronic hypoxia and positions retinal microcirculation as a sensitive indicator of systemic oxygen homeostasis. These findings establish an essential baseline for future research and may contribute to a deeper understanding of the microvascular mechanisms underlying ocular and systemic conditions in high-altitude populations.

## Figures and Tables

**Table 1:** Systemic and ocular parameters of the population studied.

Parameters	Mean ± SD
visual acuity	0.13 ± 0.22
near visual acuity	−0.15 ± 0.15
best-corrected visual acuity	0.03 ± 0.08
heart rate	91.32 ± 14.10
systolic blood pressure, mmHg	96.11 ± 10.41
diastolic blood pressure, mmHg	61.51 ± 9.71
blood oxygen saturation, %	90.44 ± 3.62
intraocular pressure, mmHg	15.26 ± 3.03
height, cm	130.11 ± 6.00
weight, kg	27.24 ± 5.47
spherical equivalence, D	0.19 ± 1.28
axial length, mm	22.78 ± 0.79

**Table 2:** Pearson correlation coefficients (R) between macular vessel density in different layers and other parameters.

Macular VD	Subfield	Systolic Blood Pressure	Blood Oxygen Saturation	Height	Weight	Spherical Equivalence	Axial Length	Subfoveal Choroidal Thickness	Foveal Thickness
SCP	superior	−0.044	0.019	0.009	0.015	0.019	−0.034	0.039	−0.003
	inferior	0.178 **	0.008	−0.019	−0.061	−0.01	−0.055	0.001	−0.042
	nasal	0.043	−0.014	−0.024	0.035	0.002	−0.014	0.04	−0.013
	temporal	0.046	−0.046	−0.019	0.005	−0.045	−0.045	0.056	−0.001
	foveal	−0.049	0.118 *	−0.001	0.053	0.037	0.075	0.056	−0.017
DCP	superior	−0.078	−0.064	0.100 *	0.065	−0.066	0.085	0.007	−0.095
	inferior	0.03	−0.102 *	0.056	0.037	−0.075	−0.004	−0.008	−0.091
	nasal	−0.078	−0.114 *	0.092	0.103 *	0.051	0.027	0.06	−0.061
	temporal	−0.075	−0.088	0.06	0.044	−0.064	0.026	0.03	−0.047
	foveal	−0.021	0.143 **	0.012	0.064	0.04	0.04	0.019	0.025
CC	superior	0.117 *	0.003	0.078	0.04	−0.028	−0.027	−0.024	0.005
	inferior	0.029	−0.004	0.055	−0.022	−0.091	−0.013	0.004	−0.059
	nasal	0.052	−0.024	0.091	0.026	−0.032	0.051	−0.049	−0.045
	temporal	0.076	−0.033	0.083	0.022	−0.057	0.068	−0.076	0.026
	foveal	−0.027	0.004	0.117 *	0.039	−0.009	−0.012	0.009	−0.052

VD, vessel density; DCP, deep capillary plexus; SCP, superficial capillary plexus; CC, choriocapillaris. * *p* < 0.05, ** *p* < 0.01.

**Table 3:** Multivariate linear analysis of factors affecting foveal vessel density in different layers.

Dependent Variable		Unstandardized Beta	Standard Error	*p*
Foveal SCP VD	sex (male)	−0.099	0.503	0.844
	axial length	0.64	0.368	0.083
	spherical equivalence	0.312	0.221	0.159
	blood oxygen saturation	0.16	0.07	0.022 *
Foveal DCP VD	sex (male)	0.045	0.488	0.926
	axial length	0.44	0.357	0.219
	spherical equivalence	0.312	0.214	0.146
	blood oxygen saturation	0.2	0.068	0.003 *
Foveal CC VD	sex (male)	0.117	0.363	0.748
	axial length	0.016	0.266	0.951
	spherical equivalence	0.082	0.16	0.61
	blood oxygen saturation	0.007	0.05	0.886

VD, vessel density; DCP, deep capillary plexus; SCP, superficial capillary plexus; CC, choriocapillaris. * *p* < 0.05.

## Data Availability

The datasets generated and analysed during the current study are not publicly available due to the personal information of the participants but are available from the corresponding author on reasonable request.

## Abbreviations

The following abbreviations are used in this manuscript:

AL	Axial Length
BCVA	Best-Corrected Visual Acuity
CC	Choriocapillaris
DCP	Deep Capillary Plexus
ETDRS	Early Treatment Diabetic Retinopathy Study
IPL	Inner Plexiform Layer
OCTA	Optical Coherence Tomography Angiography
SCP	Superficial Capillary Plexus
SE	Spherical Equivalent
SpO_2_	Blood Oxygen Saturation
VD	Vessel Density
VEGF	Vascular Endothelial Growth Factor
